# Biochemical and molecular changes in peach fruit exposed to cold stress conditions

**DOI:** 10.1186/s43897-023-00073-0

**Published:** 2023-11-13

**Authors:** Giulia Franzoni, Natasha Damiana Spadafora, Tiziana Maria Sirangelo, Antonio Ferrante, Hilary J. Rogers

**Affiliations:** 1 Department of Agricultural and Environmental Sciences, University of Milan, Via Celoria 2, 20133 Milan, Italy; 2 Department of Chemical, Pharmaceutical and Agricultural Sciences, University of Ferrara, 44121 Ferrara, Italy; 3 ENEA-Italian National Agency for New Technologies, Energy and Sustainable Economic Development-Division Biotechnologies and Agroindustry, 00123 Rome, Italy; 4 School of Biosciences, Cardiff University, Sir Martin Evans Building, Museum Avenue, Cardiff, CF10 3AX UK

**Keywords:** Chilling injury, Postharvest, Cold stress, Gene expression, Molecular tools, *Prunus persica* L

## Abstract

Storage or transportation temperature is very important for preserving the quality of fruit. However, low temperature in sensitive fruit such as peach can induce loss of quality. Fruit exposed to a specific range of temperatures and for a longer period can show chilling injury (CI) symptoms. The susceptibility to CI at low temperature varies among cultivars and genetic backgrounds. Along with agronomic management, appropriate postharvest management can limit quality losses. The importance of correct temperature management during postharvest handling has been widely demonstrated. Nowadays, due to long-distance markets and complex logistics that require multiple actors, the management of storage/transportation conditions is crucial for the quality of products reaching the consumer.

Peach fruit exposed to low temperatures activate a suite of physiological, metabolomic, and molecular changes that attempt to counteract the negative effects of chilling stress. In this review an overview of the factors involved, and plant responses is presented and critically discussed. Physiological disorders associated with CI generally only appear after the storage/transportation, hence early detection methods are needed to monitor quality and detect internal changes which will lead to CI development. CI detection tools are assessed: they need to be easy to use, and preferably non-destructive to avoid loss of products.

**Graphical Abstract:**

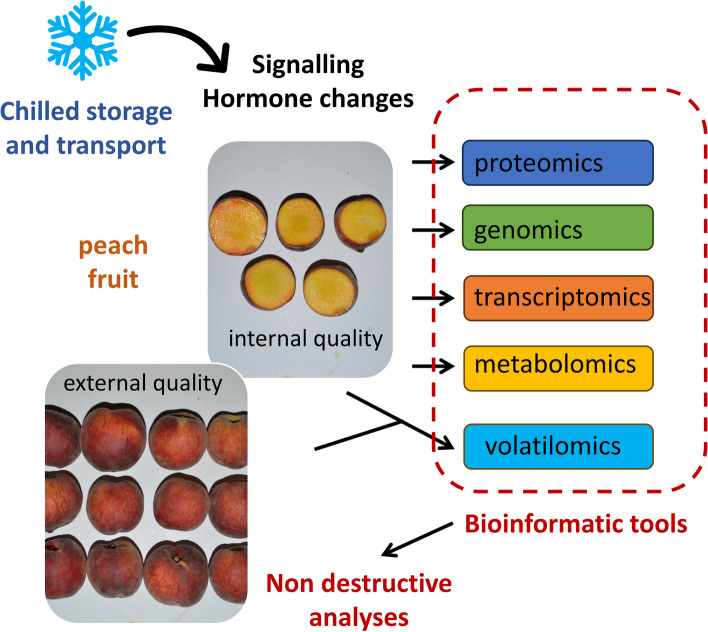

## Introduction

Fruit can be preserved by lowering the temperature during the postharvest distribution chain. Low temperature usually slows metabolic processes such as respiration and ethylene biosynthesis which are required for continued ripening, with benefits for shelf life. However, the range of temperatures that can provide positive effects depends on the species.

**Fig. 1:**
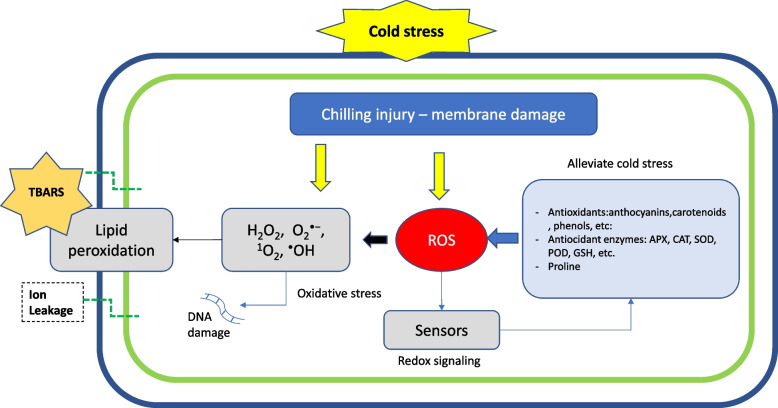
Cold stress acts on plasmalemma and cell membranes. Prolonged exposure to cold induces the accumulation of reactive oxygen species (ROS), such as O2-(superoxide radical) and H2O2 (hydrogen peroxide), acting as potential signalling molecules. ROS can reduce membrane integrity with destabilization of cellular compartments. The loss of cell compartmentalization can induce ion leakage, and several physiological disorders affecting fruit quality such as browning or loss of firmness. ROS can also induce DNA damage and the accumulation of antioxidant compounds. Redox signalling and antioxidant enzyme (APX, CAT, SOD, POD, GSH, etc.) responses are schematized, as well as membrane destabilization, due to lipid peroxidation

Many important fruit species deriving from tropical and subtropical origins, including tomatoes and bananas, as well as more temperate species such as peach and plum are poorly adapted to exposure to cold temperatures (Albornoz et al. 2022). In peach [*Prunus persica* (L.) Batsch], chilling injury (CI) can reduce storage life when fruits are subjected to low temperatures. CI is often only visible during post-storage ripening at 20 °C, after storage from 2 to 6 °C for 2 weeks or stored at 0 °C for 3 weeks or longer (Lurie 2022). CI is a disorder that develops internally to fruit and often only becomes visible when fruits are cut. CI is associated with membrane breakdown and disruption of cell membranes (Jung et al. 2023) (Fig. 1). Cold temperatures cause the lipids in cell membranes to become rigid, reducing their fluidity and affecting their integrity (Luo et al. 2015). This leads to the breakdown of the membranes and compromises their functionality. The loss of membrane integrity induces ion leakage from the cells and disrupts the balance of electrolytes within the fruit tissues (Ma et al. 2022; Wang et al. 2015). This disturbance in ion concentrations has adverse effects on various physiological processes and cellular functions. One of the major symptoms associated with CI is flesh browning; this is caused by polyphenol oxidases (PPO,) responsible for phenolic conversion into quinones which gain access to their substrates located in the vacuole when membranes are damaged. Cold stressed fruits also lose juiciness (increased woolliness) and flavour, both of which are linked to a loss of correct ripening metabolism (Lurie and Crisosto 2005). Flesh reddening or ‘bleeding’ is also associated with CI in fruit including peaches (Lurie and Crisosto 2005; Manganaris et al. 2008) and is due to an accumulation of anthocyanins.

Peach is a cold sensitive fruit, and its CI susceptibility depends on the genetic background, ripening stages, and orchard factors such as agronomic management and environmental conditions (Crisosto et al. 1997). The wide variability in CI sensitivity among peach cultivars, based on their susceptibility to exposure to 0 or 5 °C for different storage periods can be classified into three categories: CI non-susceptible and temperature insensitive; CI non-susceptible resilient to least 5 weeks storage at 0 °C but CI susceptible (< 5 weeks storage) at 5 °C (temperature sensitive) and finally CI susceptible (< 5 weeks storage) at both storage temperatures (Crisosto et al. 1999).

In this review we discuss transcriptomic, proteomic and metabolomic changes in peach fruit, providing an overview of the factors involved and the physiological disorders associated with CI. Furthermore, bioinformatics approaches that can lead to a further understanding of CI molecular mechanisms and their changes under specific treatments are discussed. Finally, non-destructive detection tools for CI are assessed.

## Transcriptional changes in chilled peach fruits

### Signalling pathways

**Fig. 2:**
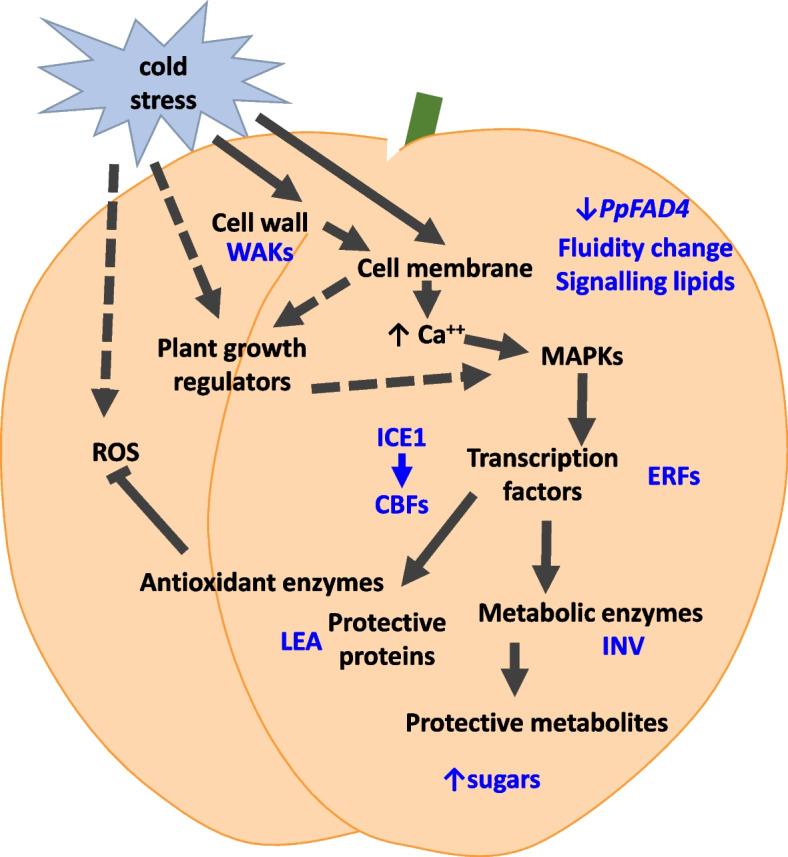
Overview of key signalling pathways for cold showing perception of the signal by the cell wall and membrane, major regulators: ICE and CBFs, other transcription factors such as ERFs that in turn regulate protective proteins and activate metabolic enzymes to produce protective metabolites. Calcium fluxes, Plant growth regulators and ROS are also involved in modulating this signalling. Arrows indicate direction of signalling with dashed arrows indicating an indirect response

Key pathways in stress signalling have been elucidated from data on a range of model and crop plants, and apply also to the responses of fruit to chilled storage. Chilling stress in plants is defined as exposure to temperatures above freezing and below the optimal temperature for the plant, typically 0–12 °C, while freezing is experienced at temperatures of 0 °C or below (Li et al. 2016). A key first target of cold stress is the cell membrane where it induces changes in the lipid membrane fluidity and composition (Jung et al. 2023) as discussed above. Changes in the levels of signalling lipids result in alteration to the cell metabolism via target proteins, localised to the cell membrane. Changes in membrane fluidity elicit changes in membrane ion channels resulting in calcium signalling (Aghdam et al. 2022). The calcium influx activates a MAP kinase signalling cascade which then switches on cold responsive genes (Zhang et al. 2022). To date, the best-characterized transcription factors involved in chilling tolerance include the C-repeat binding factors (*CBFs*), which play an important role in the acclimation of plants to chilling conditions (Hwarari et al. 2022; Shi et al. 2018). When plants are subjected to cold stress, the positive regulators *ICE1* (inducers of CBF expression 1), *CAMTA3* (calmodulin-binding transcription activator 3) and *BZR1/BES1* (brassinazole-resistant 1) and negative regulators *MYB15, PIFs* (phytochrome-interacting factors) and *EIN3* (ethylene-insensitive 3) modulate CBF gene expression (Liu et al. 2019b). Then, the CBF proteins bind to the C-repeat of downstream cold-responsive (*COR*) genes (Song et al. 2021; Fig. 2).

The cell wall may also participate in cold stress signalling through changes in pectin-remodelling enzymes and pectin-associated receptors, known also as wall-associated kinases (WAKs) (Le Gall et al. 2015). Phytohormones also mediate cold stress responses including gibberellic acid (GA), abscisic acid (ABA), jasmonic acid (JA) cytokinins, ethylene and brassinosteroids (Eremina et al. 2016). Chilling also causes an increase in reactive oxygen species (ROS) which in turn damage membranes and results in some of the chilling injury (CI) symptoms seen in peach fruit (Huan et al. 2016). ROS, such as O2-(superoxide radical) and H2O2 (hydrogen peroxide) act as potential signalling molecules, with H2O2 stimulating lipid peroxidation and oxidative stress (Huan et al. 2016). The plant antioxidant defense system helps to scavenge ROS, thus reducing oxidative damage (Fig. 1). Indeed, there was a correlation between high levels of phenolics, antioxidants or sugars with lower CI development (Abidi et al. 2015; Aghdam et al. 2020). A range of protective proteins and enzymes are involved in mitigating the effects of chilling stress (Kazemi-Shahandashti and Maali-Amiri 2018). Some of these are involved in ROS detoxification such as superoxide dismutase (SOD), peroxidase (POD), and catalase (CAT) (Zhu et al. 2022; Tang et al. 2022). SOD can convert O^2−^·into H_2_O_2_, and after this step, POD and CAT decompose H_2_O_2_ to H_2_O (Tang et al. 2022).

The Ascorbate–Glutathione (AsA-GSH) cycle is another important component of the ROS scavenging system consisting of ascorbate peroxidase (APX), glutathione peroxidase (GPX), and glutathione reductase (GR). Ascorbate peroxidase also contributes to the degradation of H_2_O_2_ (Tang et al. 2022) (Fig. 1).

Another group of proteins is produced in response to freezing, namely antifreeze proteins which are similar to pathogenesis-related (PR) proteins (Hon et al. 1995). These proteins are primarily associated with pathogen responses their expression also changes in response to postharvest cold storage. Several protein families are involved in protection of membranes and other proteins including dehydrins, and heat shock proteins which act as chaperones (Bassett et al. 2015).

There is evidence that certain *Hsfs* (Heat stress transcription factors) directly sense ROS species, such as H_2_O_2_; O_2_^−^, and regulate gene expression during oxidative stress. For example, the transcription of genes encoding the cytosolic peroxidases APX1 and APX2 can be regulated by these transcription factors, that in turn can be modulated by ROS (Hossain et al. 2015).

Thaumatin-like proteins are also accumulated during peach cold acclimation and may also protect the integrity of the plant cells (Dagar et al. 2010; Liu et al. 2010). In peach fruit a number of these genes and pathways have been associated with chilling and protection from CI (Fig. 2).

Peach cell membrane dysfunction is one of the major molecular events elicited by low temperature storage and indeed varieties more and less resistant to CI differ in their lipidome (Bustamante et al. 2018). Moreover, a higher proportion of unsaturated fatty acids was linked to reduction in peach CI stress (Zhang and Tian 2010). Fatty acid desaturases (FADs) are important in modulating membrane fluidity, and transcriptional regulation of these genes in peach has been associated with metabolic changes occurring during chilling stress conditions (Song et al. 2022). Both in several peach and nectarine [*P. persica* (L.) Barsch, var. nectarina] cultivars, *PpFAD4* gene expression was found to decrease under chilling conditions, showing very significant differences among peach varieties before and after cold storage (Muto et al. 2020).

Several tools have been developed to study the effects of chilling on peach fruit and more specifically the genes involved in regulating chilling injury, such as the ChillPeach (Ogundiwin et al. 2008) and μPEACH1.0 (ESTree Consortium 2005) expression microarrays, each containing over 4000 gene targets. Using the ChillPeach array known components of the cold response signalling pathway were identified including CBF1, many of which were common to genes identified in cold-treated Arabidopsis plants. The Chillpeach array was combined with a bulked segregant analysis (Pons et al. 2014), using groups of fruits showing extreme CI responses. A number of stress-related and phytohormone-related transcription factors and regulons identified from Arabidopsis were found to be up-regulated, including the ICE1 and CBF genes. This study indicated that extended exposure to cold which induces CI resulted in a reduced response of ICE-CBF genes but fruit with lower sensitivity to CI were able to retain expression of these genes for longer.

The overexpression of the peach gene *PpCBF1* in apple resulted in an enhancement of cold tolerance (Wisniewski et al. 2015), and of particular relevance here, expression of the *Arabidopsis thaliana CBF1* gene in tomato fruit postharvest protected the fruit against chilling injury, demonstrating its role in chilling protection in fruit (Albornoz et al. 2023). Recently it was shown that the *PpCBF6* expression increases rapidly when peach fruit are subjected to cold, alongside an increase in the expression of the *PpVIN2* gene. PpVIN2 is a cold-sensitive vacuolar invertase inhibitor, and regulator of sucrose metabolism (Cao et al. 2021). By limiting the invertase activity, *PpCBF6* keeps cellular sugar levels elevated, providing an enhanced protection to the plant under stress. It was demonstrated that *PpCBF6* binds to the promoter of *PpVIN2*, and transient over-expression of *PpCBF6* in peach fruit has a negative regulatory effect on *PpVIN2* expression. These indicate promising potential routes for improving varieties to reduce CI through conventional breeding or future biotechnological approaches.

CBF regulation of chilling responses may also be linked to the effects of exogenous applications of plant hormones. Treatment of peach fruit with JA before chilled storage reduces internal browning, one of the effects of chilling injury (Chen et al. 2019; Zhao et al. 2021a). *PpCBF6* appears to be regulated by methyl jasmonate (MeJA) (Cao et al. 2021) thus the action of exogenous JA might be via the modulation of CBF expression. Zhao et al. (2021a) found that JA induced ethylene production via the upregulation of ethylene biosynthesis gene *ACS1*. Ethylene treatment is known to reduce browning (although it increases softening) and exogenous ethylene treatment upregulated a number of lipid related genes (Zhu et al. 2019). Thus, ethylene may be acting via the modulation of lipid metabolism, perhaps increasing membrane stability. However, Zhao et al. (2021a) also found that the JA treatment increased soluble sugar concentrations in the fruit which would also have a protective effect against the cold stress. Thus, the JA may be exerting its protective effect via several complementary routes.

PpVIN2 enzyme activity is further modulated by peach invertase inhibitors (PpINHs) (Wang et al. 2020). It was found that peach fruit transiently overexpressing *PpINH1* had decreased VIN activity, thus increased sucrose content and improving the chilling stress tolerance of the fruit. Indeed, the link between sugars and chilling injury in peach fruit may relate more to the rate of sucrose degradation than the levels of sucrose at harvest. Moreover, the rate of sucrose degradation is also regulated by ethylene. Treatment of peach fruit with the ethylene signalling inhibitor 1-mythylcyclopropane (1-MCP) reduced chilling injury (Jin et al. 2011). This effect was attributed to a reduction in oxidative damage through enhancing antioxidant enzyme activity. However, the effects of 1-MCP may also be mediated by reducing sucrose degradation. In another study 1-MCP effects were more marked in a variety in which sucrose degradation was faster than a variety in which the initial sucrose concentration was higher, but the rate of its degradation was slower (Yu et al. 2017). However, results with 1-MCP seem to differ across cultivars (Yu et al. 2017). Transcriptomic studies to assess the effects of 1-MCP in different cultivars may be informative in understanding the complex effects of ethylene on chilling injury in peaches.

CBF-independent regulatory pathways have also been analysed in plants under chilling stress conditions, and in particular ABA responses are included in CBF independent pathways (Shi et al. 2015). These CBF independent pathways may also operate in chilled peach fruit as treatment with ABA reduces chilling injury (Zhao et al. 2022). This is consistent with findings suggesting that ABA reduces H_2_O_2_ content and ethylene production (Tang et al. 2022).

Both genomic level and transcriptomic level studies have been used to gain insights into how these regulatory genes and downstream protective genes might be associated with the sensitivity or resilience to chilling injury.

### Genome-wide tools to identify candidate genes for CI resilience

A QTL analysis identified nine candidate genes for mealiness in nectarine ‘Venus’ (Nuñez-Lillo et al. 2015). These included three NAC transcription factors (with homology to Arabidopsis *ANAC072, ANAC101* and *ANAC032*), one *bZIP* one AP2 domain (*ERF4*) and two *MYB-like* transcription factors. The nine genes also included a glycosyl hydrolase and a protein kinase domain gene. Some of these genes were also candidate genes for maturity date and *ANAC072* was suggested as a key gene in this pleiotropic effect of maturation date on mealiness. These two characters may be linked as it is well-known that late season fruit are more susceptible to mealiness (Lurie and Crisosto 2005; Mitchell and Kader 1989). A subsequent QTL analysis of a peach x nectarine cross (Nuñez-Lillo et al. 2019) only identified one mealiness-associated candidate gene which encodes a glucan endo-1,3-beta-glucosidase 12 on a different chromosome to the previous study and linked to cell wall remodelling. These two studies may have identified genes in the same pathway or additive pathways, which need further work to resolve. Other SNP markers associated with mealiness have also been identified through analysis of segregating populations (Martínez-García et al. 2013) and related to a bZIP family gene on yet another chromosome and a C3HC4-type RING finger family protein which may be associated with cold-stress responses. This gene may also be associated with another CI symptom, ‘flesh bleeding’. In this same study other SNPs were related to flesh browning, including SNPs associated with *AOX1A*, a gene that encodes an alternative oxidase and may be involved in protection against oxidative stress. In a different approach Pons et al. (2014) used bulk segregant gene expression analysis to identify a gene that may be upregulated to protect fruit from CI. This gene (*TT19*) is involved in anthocyanin accumulation and modulation of cell wall composition, however in this study there were no significant differences in browning or flesh bleeding in the populations analysed, thus its role needs further verification. A GWAS analysis that sought SNPs associated with browning, mealiness and bleeding (Dhanapal and Crisosto 2013) identified three potentially useful SNPs with different candidate genes for mealiness, bleeding and browning. Clearly there are links between these three CI characters: mealiness and bleeding seem to be associated symptoms which may be related to senescence (Lurie 2022; Lurie and Crisosto 2005). At a chromosomal level, LG4 seems to contain a region associated with susceptibility to develop mealiness and bleeding, with candidate genes for bleeding also located on LG1 while a region on LG5 seems to be associated with browning. However, it is still unclear whether there are common regulators of all three characters. Further work is needed to test whether candidate genes are indeed required for the development of the CI symptoms and whether this is also dependent on the peach/nectarine cultivar.

### Using transcriptomic comparisons across sensitive and susceptible cultivars

A different approach has been to use global expression studies to identify pathways and regulatory genes that are upregulated either during the development of CI or earlier, during chilled storage and which might be helpful in understanding the processes that lead to CI development. This approach has been used in a number of studies to compare differences between cultivars or peaches and nectarines that are differentially sensitive to CI. Microarray studies, based on the ChillPeach (Ogundiwin et al. 2008) and μPEACH1.0 (ESTree Consortium 2005) platforms showed that antioxidant and protective genes were more highly expressed at harvest in a cold-tolerant nectarine (cv. Yuval) compared to a more sensitive peach (cv. Oded) (Dagar et al. 2013). Using the μPEACH 1.0 to compare differentially CI susceptible peach cultivars (‘Morettini No2’ and ‘Royal Glory’). Falara et al. (2011) also identified a number of protective genes including a dehydrin, heat shock proteins as well as cell wall modification genes (an expansin and a β-D-xylosidase) which were more highly expressed in the more tolerant cv. Royal Glory. The Chillpeach microarray was also used to contrasting peach cultivars: ‘Oded’ which is CI tolerant and ‘Hermoza’ which develops browning and bleeding, but not wooliness, before symptoms develop (Puig et al. 2015). Core cold responsive genes were expressed at lower levels in the more CI-tolerant cultivar while cell-wall and pectin remodelling genes were up-regulated by the cold treatment and an increase in antioxidant-related gene expression was linked to cold-tolerance. More recently the development of RNAseq based transcriptome analysis has enabled wider analysis of global transcriptome changesan a comparison of early (more resistant to CI) and late ripening varieties (Nilo-Poyanco et al. 2019). The early ripening varieties upregulated genes related to fatty acid catabolism and ethylene biosynthesis in response to cold storage, but repressed ethylene signalling and cell wall loosening related genes. More sensitive late ripening varieties expressed antioxidant related genes more highly in response to cold as well as HSF and WRKY transcription factors. It was concluded that higher ROS in early ripening cultivars may be important in their resilience to cold, allowing correct cell wall dismantling associated with normal ripening, as well as better plastid homeostasis and a better capacity to accumulate carotenoids.

### Understanding how treatments that modulate CI affect gene expression and can inform on CI development

Comparing different storage treatments that induce CI has also been used to identify genes potentially responsible for CI development. For example, comparison of peach cv. O’Henry with or without cold storage (González-Agüero et al. 2008) using a microarray identified cell wall remodelling genes and endomembrane trafficking genes up-regulated by the cold treatment which resulted in woolly flesh. Combining transcriptomic with other omic approaches has also been useful (Sirangelo et al. 2022). For example, a network analysis of metabolite and gene expression changes based on RNAseq (Wang et al. 2017) showed that cv. Hujingmilu fruit subjected to low temperature conditioning before cold storage increased ethylene production through upregulation of biosynthetic genes and upregulated specific ethylene response (ERF) transcription factors. The low temperature treatment also upregulated cell wall remodelling genes and maintained the expression of fatty acid biosynthesis genes favouring softening rather than internal browning.

A number of studies have assessed the effects of treatments to reduce CI on global gene expression patterns. For example, molecular mechanisms involved in the effect of controlled atmosphere (CA) on CI prevention during peach postharvest ripening were analyzed in “Red Pearl” nectarines (Sanhueza et al. 2015). Transcriptomic changes indicated that low O_2_ combined with cold storage significantly slowed down the metabolic ripening processes with a reduction in ethylene related gene expression, more than cold storage alone which resulted in the development of a less mealy texture. In another study RNA-Seq was used to compare the effects of treating ‘Madoka’ peach fruit with 1-MCP pre-storage or high CO_2_ during storage at 0 °C for 12 days (Choi et al. 2021) with the cold storage alone, without pre-treatment and under standard CO_2_ levels. Both treatments reduced CI and reduced the expression of pectin solubilization-related and cell-wall disassembly-related genes as well as ethylene biosynthesis genes, thus a reduced ethylene production may be responsible for reduced weight loss seen with both these treatments. Both treatments also elicited an upregulation of stress-related genes, although genes related to JA biosynthesis appeared to be down-regulated. This seems in agreement with another study discussed above (Zhao et al. 2021a) where exogenous application of JA seemed to induce ethylene biosynthesis, however the effects on CI symptoms seem contrasting, and suggest that different components of the CI syndrome may be regulated differentially.

### Bioinformatic approaches to understand cold response in peach

Bioinformatic studies can also be helpful in exploiting existing data to understand better cold responses in peach fruit that may lead to CI. For example, using digital expression analyses of EST datasets, 164 cold-induced genes were identified involved in a range of pathways (Tittarelli et al. 2009). The promoters of three of these genes were analysed in more detail to identify important cis-elements related to cold responses. To further verify the cold-specificity of these promoters they were transiently expressed in peach fruit driving the GUS reporter gene at two temperatures. Indeed, two of them were activated by cold treatment confirming their cold-inducibility. Weighted gene correlation network analysis is also a useful tool to explore co-expression networks to try to identify upstream regulators for genes known to be implicated in CI. For example, this approach identified potential ERF regulators of CI-related genes that are up-regulated very early during chilled storage (Muto et al. 2022a). Further analysis of promoter function has also been addressed through new tools to identify motifs associated with particular expression patterns (Ksouri et al. 2021). This seems a very promising approach applied to CI to better understand the regulation of important genes able to confer CI resilience.

## Proteomic changes in response to chilling

Transcriptomes and studies of gene expression are useful to try to identify CI-associated pathways and responses in the fruit to chilling. However, the physiological effects of CI are mediated and mitigated largely by proteins. Proteomics is a useful tool to study proteins that are differentially expressed in cold stressed or chilling-injured fruit. This information can help to understand the molecular mechanisms of CI and to develop strategies to prevent or mitigate it. Various proteomic techniques, such as two-dimensional gel electrophoresis (2-DE) and mass spectrometry (MS), have been used to identify and characterize these differentially expressed proteins in chilled fruit (Wu et al. 2016). Although the detection sensitivity and identification of proteins has improved considerably through the development of better technology (Liu et al. 2019c), proteomics may be biased towards the more abundant proteins and often processes identified are similar to those identified through transcriptomics, where changes in expression of much higher numbers of genes can be detected. However, proteomic approaches are important as they demonstrate that differentially expressed transcripts are actually translated. During cold stress exposure, the biochemical and physiological changes discussed above lead both to protein accumulation and to changes in protein structure, some of which lead to subsequent cellular damage, while other proteins mitigate the stress. It is also important to note that the specific differentially expressed proteins in chilled fruit can vary depending on factors such as the duration and severity of chilling stress, variety, and experimental conditions (Neilson et al. 2010).

Several studies have used proteomics as molecular tool to study the accumulation of proteins in peach exposed to cold stress and showing chilling injury (Table 1). The use of 2-D difference gel electrophoresis (DIGE) allowed the identification of 16 proteins that were up-accumulated in peach fruit that had been maintained at 20 °C (shelf-life) versus those stored in cold storage (4 °C for three weeks) or cold room (4 °C) versus cold room storage (4 °C) plus shelf-life at 20 °C (Nilo et al. 2010). A similar proteomic study was carried out on nectarine, where half of the fruit were kept at 20 °C for 24 h (used as control) and half were pre-cooled at 0 °C for 1 h and then stored at 5 °C for five weeks. After cold storage the fruit were transferred at 20 °C for 24 h. All fruit after cold storage showed CI symptoms. Eleven proteins were differentially accumulated after cold treatment (Giraldo et al. 2012). These studies reported that the accumulated proteins were also involved in a variety of cellular processes, including cell wall metabolism, cell death, protein folding, carbohydrate, and lipid metabolism. However, there are also several proteins that decreased in expression and could be associated to CI development and appearance reduction. It is well known that protein extraction and identification depend on protocols, procedures, and instruments used. Results can also depend on the genotype of peach used for the experiment, moreover, the harvest maturity, storage temperature, and duration, as well as the recovery time can greatly affect transcription and protein translation. Many experiments should be conducted, and CI symptoms should be monitored and recorded in each proteomic study. However, many proteins identified in peach after cold stress and CI development were also identified in other chilling sensitive fruits such as tomato or melon (Song et al. 2020; Sanchez-Bel et al. 2012) and thus may be of use in understanding whether CI development has species-specific mechanisms or some shared control points.

**Table 1:** Proteins identified and accumulated under cold stress or chilling injury in peach fruit

Protein	Function	Relationship with CI	Reference
*Cell wall and membrane stabilization*			
Polygalacturonase-inhibiting protein	Regulate vacuolar invertase	Increase tolerance to cold stress protecting cell wall degradation	Wei et al. 2023
Dehydrin	Membrane stabilization	Reduce the membrane breakdown disorders	Nilo et al. 2010
Type II SK2 dehydrin	Membrane stabilization	Reduce the membrane breakdown disorders	Giraldo et al. 2012
RAD23	DNA repair protein	Recovery action against CI damage	Giraldo et al. 2012
*Plant hormone regulation*			
1-aminocyclopropane-1-carboxylate oxidase 1	Last key enzyme of the ethylene biosynthesis	CI through the membrane breakdown increases the ethylene levels	Nilo et al. 2010
*Proteins involved in the signalling network*			
Calmodulin	Calcium receptor	Stability of cell wall and cold tolerance	Dagar et al. 2010
Profilin, Pru p 4.01	Allergene	Plant defence strategy	Giraldo et al. 2012
Putative allergen, Pru du 1.06A	Pathogenesis-related protein	Plant defence strategy	Giraldo et al. 2012
Putative allergen, Pru du 1.01	Pathogenesis-related protein	Plant defence strategy	Yu et al. 2015
Pathogenesis-related like protein 1	Pathogenesis-related protein	Plant defence and involved in hypersensitive response or systemic acquired resistance	Yu et al. 2015
*Antioxidant systems and ROS scavenging*			
Catalase (CAT)	Detoxification enzyme	Hydrogen peroxide catabolic process	Giraldo et al. 2012
Gluthatione peroxidase	Scavenger activity	Response to ROS accumulation	Giraldo et al. 2012
Putative protein disulfide-isomerase	Alleviate the chilling injury by reducing ROS	Scavenger of radicals in particular hydrogen peroxide	Dagar et al. 2010
Small heat shock protein 1	Stress response and chaperon function	Prevent protein denaturation	Pavez et al. 2013
Thaumatin-like protein 1 and 2	Associated to chilling injury	unknown	Dagar et al. 2010
Thioredoxin	Redox homeostasis	Putative antioxidant function in CI damaged fruit	Nilo et al. 2010

### Maintaining protein and membrane stability

Heat shock proteins (HSPs) are associated with thermal stress, high or low temperature in plants. Chilling stress often leads to the upregulation of heat shock proteins, such as HSP70 and HSP90. These proteins play a crucial role in protecting cells from damage and maintaining protein homeostasis under stress conditions (Lurie 2022). Proteins associated with cell membrane stability, enolase, temperature-induced lipocalin, major allergen Pru p 1, and type II SK2 dehydrin were accumulated in peach (cv. Hongtao) stored at 0 °C compared with 5 °C (Zhang and Tian 2010). Fruit stored at 5 °C showed CI after 21 days, while fruit at 0 °C did not show any CI symptoms, suggesting the protective role of accumulated proteins. On the contrary, three proteins cinnamyl-alcohol dehydrogenase 5, cinnamyl-alcohol dehydrogenase 1, and chorismate mutase, related to phenolic compound metabolism were downregulated in peach at 0 °C as compared with 5 °C (Zhang and Tian 2010). The reduction of these proteins of the phenylpropanoid pathway may explain the lower browning appearance in fruit stored at 0 °C. Conversely, higher concentration of these proteins at 5 °C was positively correlated with the concentration of phenolic compounds.

CI tolerant peach “Oded” after cold storage (5 °C for 3 weeks plus 3 days at 20 °C) showed the accumulation of dehydrins and a putative disulfide-isomerase protein. Disulfide-isomerase is involved in cold protection acting on protein folding and hydrogen peroxide scavenging (Dagar et al. 2010; Kosová et al. 2023). Dehydrin proteins have been found to be accumulated under cold stress (Nilo et al. 2010). These proteins participate significantly in membrane stabilization in cells under abiotic stresses. The increase of dehydrin proteins can increase plant tolerance against cold stress (e.g. Liu et al. 2015). However more studies are needed to exploit this information at practical level, to protect fruit against chilling injury.

### Proteins involved in ROS scavenging

Cold conditions can enhance the accumulation of free radicals activating *ex-novo* translation of antioxidant enzymes and plant hormone biosynthesis or accumulation. Among ROS forms, H_2_O_2_ is a ROS that has been associated with the regulation of cell response and chilling injury intensity and a reduction in H_2_O_2_ concentration can prevent cold damage. An important role has been found to be played by the plant hormones. For example, abscisic acid (ABA) is a modulator of H_2_O_2_ and ethylene production. Peaches treated with ABA showed a delay of CI appearance by two weeks (Tang et al. 2022). Similar results have been observed with salicylic and jasmonic acids, that applied to peach fruit reduced the internal browning and H_2_O_2_ concentration by inhibiting CAT and POD enzymes (Zhao et al. 2021a).

Similar results have been observed with salicylic and jasmonic acids, that applied to peach fruit reduced the internal browning and H_2_O_2_ concentration by inhibiting CAT and POD enzymes (Zhao et al. 2021a).

Proteomic studies of cold stressed peach fruit showed an accumulation of superoxide dismutase (SOD, EC1.15.1.1), catalase (CAT, EC.1.15.11), and peroxidases (POD, EC.1.11.1.7). In particular, a proteomic study performed on peach exposed to 0 °C, CAT and glutathione peroxidase enzymes were accumulated (Giraldo et al. 2012), suggesting their role in preventing CI symptoms. In the same study, fruit stored at 5 °C showing CI reported a decrease of CAT enzyme. These enzymes help scavenge reactive oxygen species (ROS) and mitigate oxidative stress caused by chilling injury. ROS usually increases the lipid peroxidation inducing cell membrane damage that can lead to the loss of cellular compartments with browning development induced by phenol polymerization.

### Changes in cell wall remodelling enzymes

A barrier to abiotic stresses is provided by the cell wall and related proteins, which also contribute to signalling via WAKs as outlined above. In fruit, changes to the cell wall are also a key component of normal fruit ripening. Low temperatures can lead to changes in the expression of cell wall-related proteins, including expansins, pectin methylesterases, and xyloglucan endotransglucosylase/hydrolase (XTH) enzymes. These proteins are involved in cell wall remodelling and may contribute to the softening and cell breakdown observed in chilled peaches (Zhang et al. 2023). A CI study performed on peach demonstrated that endopolygalacturonase, CAT, NADP-dependent isocitrate dehydrogenase, pectin methylesterase, and dehydrins were differentially accumulated between control and chill injured fruit (Nilo et al. 2010). These results highlight the role of cell wall integrity and water status of the tissues after CI exposure. In particular, mealiness has been associated with abnormal cell wall dismantling during cold storage and the subsequent ripening (Lurie 2022).

Peach stored at 5 °C for three weeks showed the accumulation of two peptides with molecular masses comprised from 18 to 60 kDa. These peptides were found to be precursors of thaumatin-like protein 1 and 2 (Tab. 1). These proteins have been found to be associated to CI tolerance and located in the cell wall (Dagar et al. 2010). Although thaumatin-like protein expression has been widely reported in cold stressed plant tissues (Liu et al. 2010), their exact function in abiotic protection is not clear (de Jesús-Pires et al. 2020) and requires further investigation.

### Energy, sugar metabolism and protection

Glycolysis-related enzymes can have an important role to increase tolerance to chilling. These enzymes are involved in maintaining metabolic activity and providing sugars that may have a protective function in osmoregulation, cryoprotection, ROS scavenging (Van den Ende and Valluru 2008) and in signalling under cold stress (Wang et al. 2013). Key enzymes affected by CI are fructose-bisphosphate, aldolase, and enolase (Zhang et al. 2010). These enzymes play a role in energy metabolism and carbohydrate breakdown. Vacuolar invertases have an important role under chilling temperatures. In peach fruit during cold storage, 22 proteins were identified that interact with vacuolar invertase 2. In addition, the polygalacturonase-inhibiting protein has been found to be a key regulator of sugar metabolism and CI tolerance (Wei et al. 2023). The role of sugars could be associated with the osmotic potential and activation of physiological processes that accumulate cold-protectant metabolites.

### Signalling networks

An analysis of the proteome during fruit chilling has also been useful in exploring signalling pathways. Cold storage of CI tolerant peach “Oded” showed the accumulation of calmodulin (Dagar et al. 2010). Calmodulins are calcium receptor proteins that act in signaling and regulate several stress responses (Table 1). These proteins interact with COR genes and are responsible for cold acclimation and tolerance in plants (Yang et al. 2010). Calmodulin-binding transcription activators (CAMTA) are sensitive to calcium signals and calmodulin proteins play important role in cold or chilling stress (Iqbal et al. 2022). Hence a better understanding of calcium signalling in CI development would be worthy of further investigation.

Pathogenesis-related (PR) like proteins are a diverse group of proteins associated with biotic stresses. Although these proteins are involved in defence responses against pathogens, several PR-proteins have also been identified as up-regulated during abiotic stresses (Ali et al. 2018), although their role remains unclear. In particular, an accumulation of PR-5 and PR-10 proteins was found in chilled peach (Giraldo et al. 2012). Moreover, a PR-4B precursor gene was also identified as more highly expressed in a more chilling-tolerant peach cultivar (Royal Glory) and it was suggested that it might be responsible for its chilling tolerance (Falara et al. 2011). However, the accumulation of some PR proteins in stored fruit may relate to biotic defence. For example, PR-1 proteins accumulated more in peaches stored at 10 °C than at 5 °C (Yu et al. 2015) and it was suggested that this might be associated with increased decay. Thus, the function of PR proteins in various abiotic stresses such as cold or CI is still unknown and further investigation with a wide range of storage under chilling or non-chilling temperatures is needed. Functional genomics could be useful for revealing their specific role in fruit sensitive to CI.

## Metabolomics of peach exposed to chilling temperatures

Metabolomics is the study of small molecules, known as metabolites (< 1500 Da). They are defined as the intermediates or end products of several metabolic pathways that are involved in energy production, signalling, and regulation of biological processes. Metabolites are responsible for the appearance, flavour, taste, and aroma of fruits. Indeed, it is reported that there is a better correlation between phenotypic traits with metabolomic profiles than with other omics analyses (Colantonio et al. 2022). Moreover, metabolites are involved in stress response activities and stress resistance. By studying the metabolic changes occurring in fruits when exposed to abiotic stress, metabolomics aims to better understand the complicated and underlying processes in response to a specific system. Even though metabolomics is considered one of the most complicated techniques, its application has become more and more important to improve and guide the research to optimize crop production and improve plant breeding. The most common techniques used in metabolomic analysis are represented by mass spectrometry (MS), nuclear magnetic resonance spectroscopy (NMR), and chromatography. The large datasets generated through these techniques require an appropriate elaboration using statistic and bioinformatic tools in order to identify patterns, correlations, and create a sort of metabolic fingerprint associated with specific abiotic stress conditions. Moreover, the integration of metabolomic with other omics techniques may be useful to identify specific molecular markers associated with the stress and therefore predict the damage.

Fruits contain numerous metabolites, generally divided into primary and secondary metabolites. The first class includes metabolites considered the building blocks to produce bigger molecules, such as sugars, proteins, and lipids. The second group, such as flavonoid and antioxidant molecules as well as volatile organic compounds (VOCs) have a role in helping plants in response to stressful conditions. Some of these metabolites have shown an increase or a decrease in response to chilling stress in fruits.

### Sugars and cold stress

Soluble sugars, including fructose, sucrose, and glucose are one of the most studied class of metabolites associated with CI. Besides their significant role in the determination of fruit flavour, sugars play significant roles by providing the energy source in fruit metabolism. They help fruit to counterattack CI by lowering the freezing point and acting as cryoprotectants, reducing fruit dehydration through their accumulation as osmolytes, stabilizing cell membranes and preventing ion leakage. They also act as antioxidant compounds against reactive oxygen species that accumulate in fruits in response to abiotic stress conditions. It is established that sugars are involved in plant responses to cold stress, however, their concentration and composition affect fruit resistance to cold temperatures in different ways, both before harvest and postharvest. In particular, peach fruit with higher concentration of sugars are more resistant to CI than those with lower concentrations (Brizzolara et al. 2018; Tanou et al. 2017; Wang et al. 2013; Wu et al. 2016; Yu et al. 2015; Yu et al. 2017; Zhang et al. 2020) and in turn CI causes a decrease of sugars in fruit (He et al. 2018; Monti et al. 2019). The link between sugar metabolism and CI has been also reported in fruits such as pear, banana, tomato, bell pepper, and pomegranate (Delgado‐Vargas et al. 2022; Gómez et al. 2009; Liu et al. 2019a; Lorente-Mento et al. 2023; Xu et al. 2023; Yang et al. 2023). Several studies reported how the increased tolerance to CI observed in fruits in response to the application of exogenous treatments was connected to the enhancement of sugar metabolism (Zhang et al. 2021). For example, heat treated peaches showed an increase in fucose, raffinose, maltitol, glucose, isomaltose, sucrose, and sorbitol, and at the same time they showed less CI symptoms (Lurie 2022). Thermal postharvest technologies such as hot air and hot water treatments can improve fruit quality and resistance to CI also increasing the abundance of sugars in fruits. The composition of the sugars, involved in CI tolerance also depends on cultivar and ripening stage. Indeed, ‘June Gold’ peaches preconditioned at 20 °C showed less CI damage than those without preconditioning, and the concentration of xylose was higher (Tanou et al. 2017). On the contrary, Bustamante et al. (2016) found that peach cultivars that were more susceptible to CI had a higher concentration of xylose compared to the others. In another study, comparing three peach cultivars, the levels of xylose, fucose, sorbitol, and raffinose increased during cold storage regardless of the fruit sensitivity to CI (Brizzolara et al. 2018). Similarly, high concentration of glucose and fructose were found in apricot fruit with an enhanced tolerance to CI (Wang et al. 2016). Olmedo et al. (2023) performed a non-targeted metabolomic analysis by GC–MS to understand the changes in metabolite abundance in nectarine and their link with mealiness induced by cold storage. The study on primary metabolism revealed that in ripe nectarine fruit, cold storage disturbed sugar metabolism leading to the accumulation of different amino acids, organic acids, and sugars in mealy and juicy nectarines (Olmedo et al. 2023). Additional exogenous treatments have been shown to induce an increase in the accumulation of sugars in fruits and thus enhance CI resistance. Peach fruit treated with hot air and MeJA had higher sucrose concentration if compared with non-treated sample, and the combination of the two treatments was effective in alleviating CI during cold storage (Yu et al. 2016). Similarly, Zhao et al. (2021a) showed that the exogenous application of Jasmonic Acid alleviates chilling injury and reduces the internal browning of peach fruit by promoting sugar and ethylene metabolism. Other exogenous treatments such as 1-methylcyclopropene (1-MCP), methyl salicylate, 24-epibrassinolide (EBR), glycine betaine (GB), and oxalic acids resulted in increasing sucrose content and chilling tolerance in peach fruit by activating energy metabolism (Wang et al. 2019; Yu et al. 2017; Zhang et al. 2023). Thus, although an association between increased sugar content and CI appears in several studies it is not universal, and the specific sugars reported vary. Thus, further work is needed to assess whether the reduction in CI is related to specific sugars or is a more general effect.

### Lipid changes and role under cold stress

In addition to sugars’ role in chilling resistance, lipids play a crucial role in fruits in particular during postharvest storage and transportation since they are the major component of the cell membranes. As discussed above, exposure of peach fruit to low temperatures results in membrane damage causing permeability changes and affecting cell integrity. The measurement of loss of cell membrane integrity is often made by the analysis of electrolyte leakage and lipid peroxidation. Lipids are also important in the maintenance of membrane fluidity and thus involved in the protection against damage caused by chilling stress. Moreover, lipids may act as antioxidant compounds in the protection of fruit cells from the production of ROS caused by stress. Like sugars, lipids are also a good energy reserve during the stressful period, helping metabolic recovery. Song et al. (2022) recently reported that storage at a temperature of 0 °C delays the occurrence of CI in peach fruit by delaying the degradation of phospholipids, the upregulation of fatty acid desaturase and the unsaturation of fatty acids. In contrast, 4 °C storage induces the occurrence of CI. In another study the authors focused on the study of membrane lipid metabolomics in peach fruit in response to exogenous application of γ-aminobutyric acid (GABA) (Song et al. 2023). The treatment was able to improve peach tolerance to low temperature by inhibiting phospholipid degradation and thus reducing the accumulation of phosphatidic acid and maintaining higher levels of phosphatidylcholine and phosphatidylethanolamine. The accumulation of diacylglycerol and triacylglycerol in GABA-treated fruit resulted in a delayed CI and in a better protection of peach fruit. Different studies have shown that the exogenous application of brassinosteroids helps in the reduction of CI. Zhao et al. (2021b) focused on the study of the mechanism by which nitric oxide (NO) alleviates CI in peach fruit through regulating lipid metabolism. The NO treatment showed a positive effect, decreasing MDA levels, electrolyte leakage, lipoxygenase activity, and browning index. The application of melatonin at 0.1 mM induced a delay in the development of CI in peach fruit stored at 1 °C for 28 days. Melatonin is able to prevent the membrane lipid peroxidation and maintain a high ratio of unsaturated fatty acids (Gao et al. 2018). The positive role of melatonin in peach fruit has been explained by its role in the induction of hydrogen peroxide content at the beginning of storage and its inhibition thereafter. Moreover, an up-regulation of the expression of the genes involved in antioxidant responses has been observed in peach fruit (Cao et al. 2018). The useful application of melatonin treatment in alleviating CI damages in peach fruit during storage has been confirmed by other authors (Gao et al. 2018). Recently, the cold shock treatment (0 °C for 10 min) revealed to be useful in alleviating chilling injury in peach fruit by regulating antioxidant capacity and membrane lipid metabolism (Ma et al. 2022). In particular, peaches showed delayed CI symptoms, low EL and MDA content, and they maintained a high ratio of unsaturated membrane fatty acid to saturated fatty acids.

In addition to their role in cell membranes, lipids are also an important component of plant cuticle that cover the surface of fruit. The plant cuticle acts as an external shield safeguarding fruit from the surrounding environment, and it serves essential roles in both fruit growth and development. Currently, there is significant interest in understanding its impact on postharvest fruit quality and research indicates that the cuticle plays a pivotal role in determining crucial traits for shelf life and storage potential, such as water loss, fruit dehydration, vulnerability to rot, pests, disorders, and even firmness (Lara et al. 2019). A recent study aimed to investigate the effect of the application of calcium chloride to alleviate chilling injury in peach fruit during low-temperature storage (Ali et al. 2021). The authors observed a positive effect of the treatment with a reduction of the rate of fresh weight loss, probably related to a better cell turgidity, a decrease of fruit respiration, or a less disintegration of the fruit cellular structure. However, the role of cuticle in peach fruit during the postharvest is not completely clear. Belge et al. (2019) indagated the effect of heat and CO_2_ treatment before storage in peach fruit. They observed that peach fruit had an increased amount of cutin, but not waxes, after cold storage. Their results suggest that water loss in peach fruit may be caused by a combination of factors, which encompass not only the amount of cuticle present but also its structure, as well as the proportions of different compounds or types of compounds within it.

### Amino acid changes

The metabolism of amino acids contributes to mitigating CI. Jia et al. (2022) investigated the role of glycine betaine (GB) in the mitigation of CI in peaches during cold storage, and its effect on flavour quality through physiological and metabolomic methods. The sweetness of treated fruit was higher during storage, and this might be related to the contributions of TSS and some amino acids. Indeed, treated fruit showed higher concentration of proline, polyamines and γ-aminobutyric acid (GABA) than non-treated fruit, suggesting a positive role of GB treatment both on cold tolerance and fruit quality. Higher levels of amino acids accompanied by reduced CI and MDA levels, have been reported in peach fruits in response to hot water treatments the result of the activation of the biosynthesis of amino acids and suppression of their degradation (Wang et al. 2021). Reduced CI and MDA content in peach fruit treated with hot water were accompanied with higher levels of amino acids, phenolic compounds, and their derivatives contents such as arginine, proline, γ-aminobutyric acid (GABA), polyamines (PAs), cholorogenic acid, kaempferol and quercetin if compared to control fruit. Arginine is one of the most versatile amino acids, playing different roles in plant cells, as a building block of proteins, but also as a precursor for the synthesis of proline, GABA and PA. Therefore, the accumulation of amino acids in response to hot water treatment might play a significant role in fruit metabolism and chilling signal during cold storage. Proline plays important roles in increasing cellular osmolarity, stabilizing membrane and subcellular structures, and protecting cells against oxidative damage.

## Non-destructive analyses for monitoring chilling injury

Several non-destructive techniques have been used to assess and investigate CI in fruits, aiming to detect defects including mealy/woolly texture or browning. These methods can be classified into three groups: mechanical methods, spectroscopic and combined methods.

Acoustic, impact, ultrasonic and vibration, are common mechanical methods used in fruit firmness determination (Cakmak 2019).

Variations in internal quality such as ripeness, firmness, bruising, browning, and mealiness, can be easily monitored with Nuclear Magnetic Resonance (NMR) and Magnetic Resonance Imaging (MRI), effective but still quite expensive techniques. Changes in the chemical composition and integrity of cellular structures influence the movement of water in the plant tissues, which can be detected as proton “relaxation” in water and monitored with MRI. An NMR profiling approach was applied to investigate variations in metabolic profile and metabolite content in highly consumed crops including peaches (Santucci et al. 2015). Changes were evaluated during three subsequent collections and cold storage of fruit samples coming from a local farm and on a large-scale distribution. Peaches from a local farm exhibited a great variation in the metabolic fingerprint in relation to collection time and cold storage. The large-scale distribution peaches showed similar metabolic profiles between them all (Santucci et al. 2015).

Visible/near-infrared (vis/NIR) spectroscopy is another spectroscopic method used to monitor CI in fruit. Molecules exposed to radiation with a light source absorb the light at particular wavelengths. The absorption bands observed in the infrared region appear in the NIR spectral range. This provides subtle data about the chemical composition of fruits (Cakmak 2019). This technique uses a non-destructive index based on the difference of absorbance between two wavelengths near the absorption peak of chlorophyll-a, and it allows monitoring of changes in peach ripeness processes (DA index) (Sortino et al. 2021).

A non-destructive technique using vis/NIR spectroscopy was applied to develop a prediction model of peach flesh firmness and compared with a second prediction model developed to measure water-soluble pectin content (Uwadaira et al. 2018). The use of this technique and NMR spectra suggested that absorptions of methanol and succinate as well as galacturonic acid play a key role in peach flesh firmness prediction (Uwadaira et al. 2018). This multi-platform approach could be of use in non-destructive VIS–NIR prediction model applications.

The Vis-NIRS technique was also applied for the rapid assessment of peach internal quality and maturity in the field by evaluating how the rootstock vigour and fruit developmental stage are influenced by preharvest factors (Minas et al. 2023). Variable genotypes provided a peach fruit population with different physicochemical properties for an adequate Vis-NIRS-based model calibration. The prediction model based on the regression statistical approach showed that dry matter content, and soluble solids concentration, and index of absorbance difference could be accurately estimated with a single scan during fruit growth and maturation.

Hyperspectral imaging integrates spectroscopy and digital imaging techniques to provide data about inner and outer quality of the fruit.

Hyperspectral imaging has been used to detect cold injury in peach, and an ANN (Artificial neural network) model was developed as a supervised learning network for prediction and classification (Pan et al. 2016). Here, chill-damaged peaches showed significant loss in fruit quality compared to normal fruit, in terms of firmness, soluble solids content, extractable juice, titratable acidity and chlorophyll content, and the overall classification accuracy of chill damage was 95.8% for all samples stored in cold conditions (Pan et al. 2016).

Hyperspectral reflectance imaging combined with chemometrics allowed the evaluation of CI in peaches. To date, several machine learning models have been used to predict peach maturity using non-destructive data, however only few comparisons of these models have been carried out. Discriminating classification models, including partial least squares-discriminant analysis (PLS-DA), ANN and support vector machines (SVM), were adopted for two (non-chilled and chilled), three (non-chilled, semi-chilled and heavy-chilled) and four (non-chilled, slight-chilled, moderate-chilled, and heavy-chilled) peach classes (Sun et al. 2017). The ANN models showed the highest prediction rates, with an accuracy of higher of 85.0% for all class classifications. Overall, the results suggest that hyperspectral reflectance imaging combined with chemometrics may be suitable for the non-destructive detection of CI in peaches (Sun et al. 2017).

Hyperspectral imaging technology was applied for the quality detection of nectarines through machine learning models (Huang et al. 2021). Soluble solid content was measured as an internal quality index while the external quality indices included: intact, cracked, and dark damage. PLS, Least Squares support vector machines (LS-SVM) and Extreme Learning Machine (ELM) methods were used to determine the external quality discrimination and internal quality prediction models. Accuracies above 85% were obtained in the identification of the external quality for all methods used with the LS-SVM showing the highest identification at 94.5%. Determination coefficients and root means squared errors predicted SSC with best fit for LS-SVM. LS-SVM was indicated an excellent potential model to predict and discriminate the internal and external quality of nectarines (Huang et al. 2021).

Recently, an artificial neural network (ANN) excelled above other models for the prediction of peach maturity based on a given dataset of non -destructive data, with the linear discriminant analysis ranking second, followed by logistic regression, gradient boosting machine, random forest, SVM, and k-nearest neighbours (Ljubobratović et al. 2022).

Xuan et al. (2022) used hyperspectral imaging to evaluate inner and outer quality of a peach cultivar, by providing spectral as well as spatial data. Mask-image was used to segment the fruit region for peach solids content and firmness evaluation, together with pixel size and area for diameter and weight evaluation. Multiple linear regression models were developed, and the highest prediction coefficient was for weight (0.957), followed by soluble solid content (0.841) and firmness (0.826). The overall results showed that this technique provided a holistic approach to develop classification systems for quality level identification in peach (Xuan et al. 2022).

Several studies, evaluating the potential of the electronic nose (e-nose) as instrument to acquire volatile compounds (VOCs) non-destructively have demonstrated that this technique can be used successfully to predict parameters such as total soluble solids, pH, and firmness of fruits at different maturity stages, as well as to assess ripening (Sanaeifar et al. 2016; Srivastava and Sadisatp 2016). Wei et al. (2018) applied this technique to detect decay in peach fruit during the cold chain (0 °C). Yang et al. (2020) detected peach VOCs and the e-nose technology was applied to predict the levels of compression damage in this fruit, to discriminate between damaged and intact fruit and the elapsed time. The accuracy rate for damaged fruit identification was 93.33% 24 h after damage. The combined use of e-nose and gas chromatography-mass spectrophotometry (GC–MS) showed a change in VOC profiles after being compressed, thus this technique is promising for monitoring compression damage in peach. However, the drawback of the e-nose approach is that it is not linked to specific identifiable compounds, therefore further work is required to assess its robustness with a wide range of varieties, and across different seasons.

VOC profiles across three peach and three nectarine cultivars grown under the same environmental conditions before and after storage were compared in a recent study (Muto et al. 2020), using an enhancement of thermal desorption gas chromatography time of flight mass spectrometry (TD-GC-ToF–MS), where VOC extraction was performed from the whole fruits, for all cultivars. Here, VOC profile, gene expression and changes during cold storage were shown to be cultivar-specific, and consequently potential markers for cold storage may be obtained from a given VOC dataset. However, VOC profiles can change across seasons even for fruit of the same variety grown in the same location (Baldwin et al. 2023; Spadafora et al. 2019) hence further work is needed to assess VOC profiles to identify robust markers.

Peach quality changes are also assessed through aroma, physical characters, and sensorial features (Muto et al. 2022b). Here, six peach and nectarine cultivars were sampled at harvest and after 7 days of 1 °C storage. The different analyses revealed interesting correlation patterns, and the cultivars showed different cold storage responses. Sensory parameters were correlated with VOCs and intrinsic characters, like acidity, firmness, harmony, sweetness, bitterness, astringency, and crunchiness. This is important as aroma perception does not map fully onto instrument values, thus the use of VOC markers for assessment of aroma loss during cold storage and as a result of CI should also take account of sensory data.

Overall, many studies reported the use of vis/NIR and hyperspectral imaging for the evaluation of fruit quality attributes, such as soluble solid content, firmness, extractable juice, in many species, including peach. To date, it is evident that these techniques are the most popular ones to evaluate and monitor CI in fruits.

However, techniques based on VOC analysis of the whole fruit may also be of value as they too are non-destructive and can provide additional information on aroma loss and could be used as quality markers for fruits subjected to cold storage.

## Conclusion

Research studies related to CI have been provided important knowledge for improving quality preservation after harvest. The molecular mechanisms behind the physiological and biochemical responses can help in the selection of genotypes that could produce more tolerant fruit. A key role seems to be played by the plasmalemma and cell membrane in maintaining cell functionality. Specific breeding programmes can be planned to enhance the quality retention during storage or long-distance transportation. Low temperatures provide several benefits including longer shelf-life and ethylene production unless fruits are sensitive to CI. The improvement of knowledge associated with cold storage of CI sensitive fruit could help in the selection of the best storage facilities and technologies with potential benefits on product marketing and consumer quality assurance.

## Data Availability

Not applicable.

## Abbreviations

1-mcp: 1-Mythylcyclopropane
2-de: Two-dimensional gel electrophoresis
Aba: Abscisic acid
Ann: Artificial neural network
Asa-gsh: Ascorbate-glutathione
Apx: Ascorbate peroxidase
Bzr1/bes1: Brassinazole-resistant 1
Camta: Calmodulin-binding transcription activator
Cat: Catalase
Cbfs: C-repeat binding factors
Ci: Chilling injury
Ca: Controlled atmosphere
Dige: Difference gel electrophoresis
Ein3: Ethylene-insensitive 3
Elm: Extreme learning machine
Erf: Ethylene response transcription factors
Fads: Fatty acid desaturases
Ga: Gibberellic acid
Gaba: γ-Aminobutyric acid
Gb: Glycine betaine
Gc-ms: Gas chromatography-mass spectrophotometry
Gpx: Glutathione peroxidase
Gr: Glutathione reductase
H_2_o_2_: Hydrogen peroxide
Hsfs: Heat stress transcription factors
Hsps: Heat shock proteins
Ja: Jasmonic acid
Ls-svm: Least squares support vector machines
Ms: Mass spectrometry
Meja: Methyl jasmonate
Mri: Magnetic resonance imaging
Nmr: Magnetic resonance spectroscopy
No: Nitric oxide
O_2_-: Superoxide radical
Pls-da: Partial least squares-discriminant analysis
Pr: Pathogenesis-related proteins
Pifs: Phytochrome-interacting factors
Pod: Peroxidase
Ros: Reactive oxygen species
Sod: Superoxide dismutase
Svm: Support vector machines
Td-gc-tof-ms: Thermal desorption gas chromatography time of flight mass spectrometry
Vis/nir: Visible/near-infrared
Vocs: Volatile organic compounds
Waks: Wall-associated kinases
Xth: Xyloglucan endotransglucosylase/hydrolase
